# Supplementary material for: Carbon Quantum Dots Modified (002) Oriented Bi2O2CO3 Composites with Enhanced Photocatalytic Removal of Toluene in Air
Source: Nanomaterials (Basel). 2020 Sep 9;10(9):1795. doi: 10.3390/nano10091795 (PMC7559015; doi:10.3390/nano10091795)
Supplement: Supplementary file 1 [file nanomaterials-10-01795-s001.pdf]

nanomaterials

MDPI

Supplementary Materials

Carbon Quantum Dots Modified (002) Oriented $Bi_2O_2CO_3$ Composites with Enhanced Photocatalytic Removal of Toluene in Air

Junping Ding [1,2], Huanchun Wang [3,*], Yidong Luo [1], Yushuai Xu [1], Jinsheng Liu [2], Ruichu Lin [2], Yuchen Gao [2] and Yuanhua Lin [1,*]

[1] State Key Laboratory of New Ceramics and Fine Processing, School of Materials Science and Engineering, Tsinghua University, Beijing 100084, China; djp15@mails.tsinghua.edu.cn (J.D.); ydluozd@163.com (Y.L.); xuyushuai5736@163.com (Y.X.)

[2] China Astronaut Research and Training Center, Beijing100094, China; goldsix@sohu.com (J.L.); linruichu@163.com (R.L.); accgao@163.com (Y.G.)

[3] High-Tech Institute of Xi'an, Xi'an, Shanxi 710025, China

* Correspondence: wang-hc12@tsinghua.org.cn (H.W.); linyh@mail.tsinghua.edu.cn (Y.L.); Tel.: +86-10-6277-3741 (Y.L.)

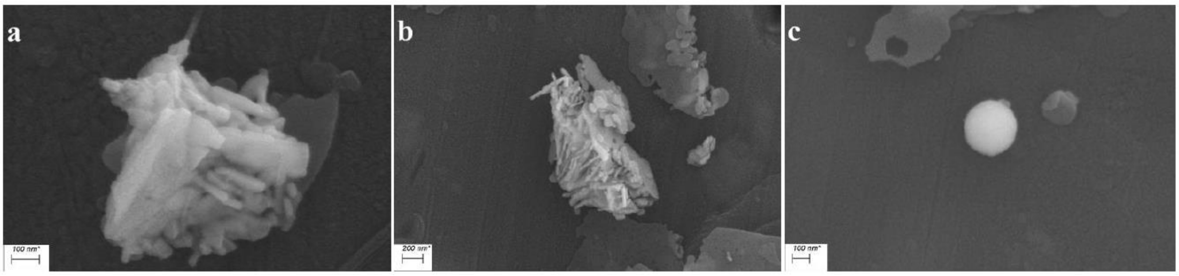

Figure S1. SEM images of BOC **(a)** and BOC-CQD-10 **(b,c)**.

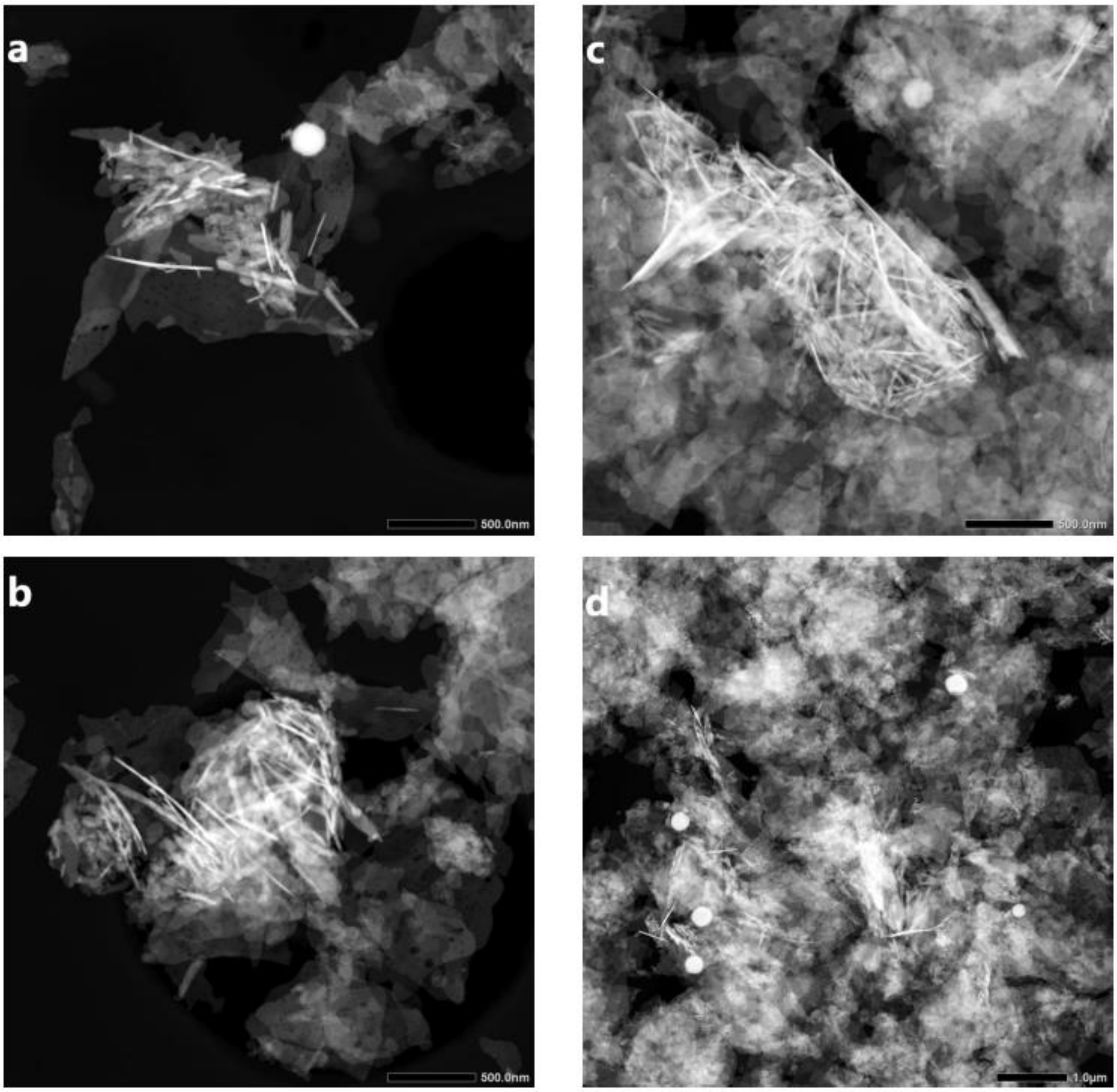

Figure S2. TEM images of BOC-CQD-15 (a, b, c, d).

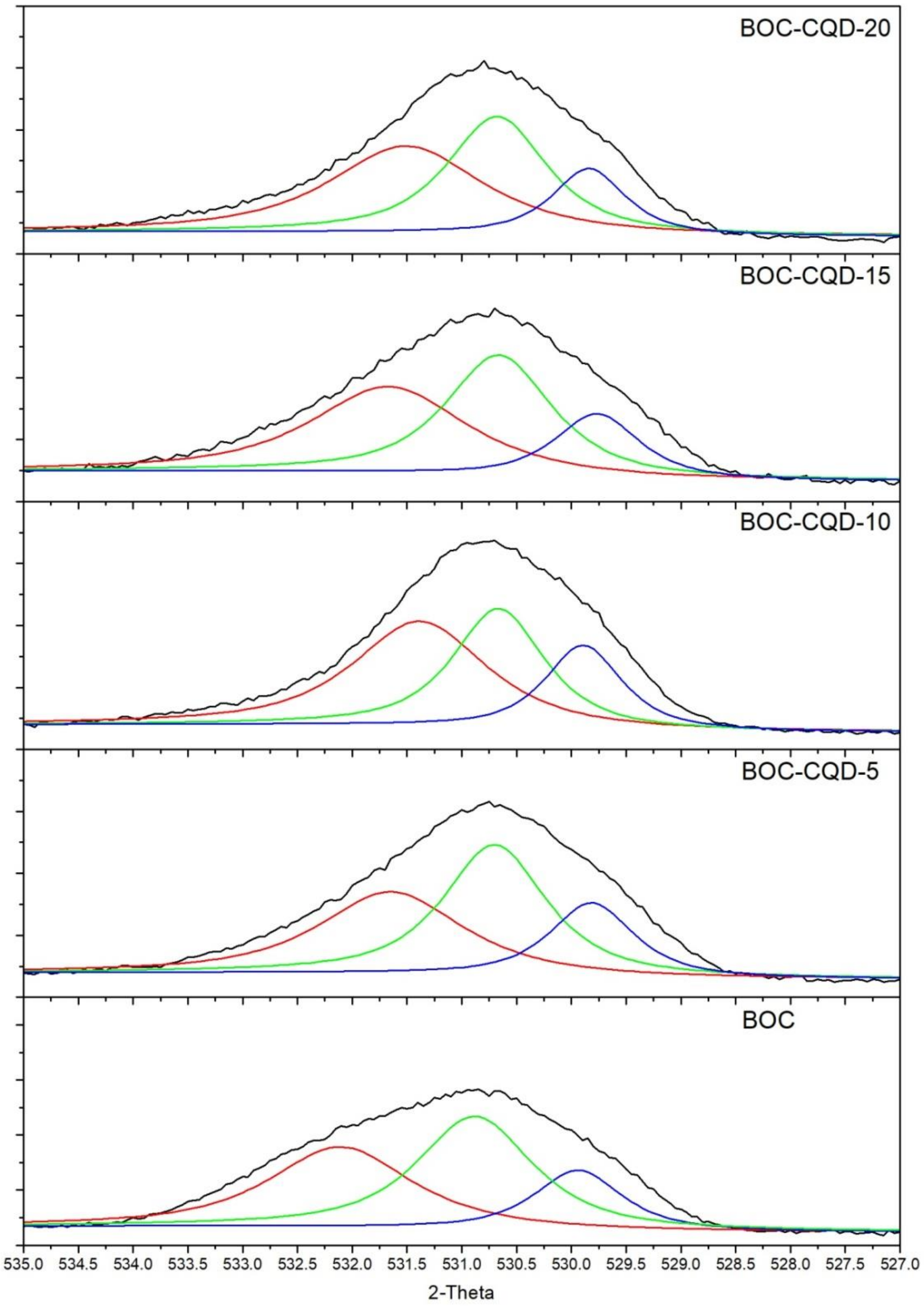

Figure S3. XPS spectra of BOC samples: O1s.

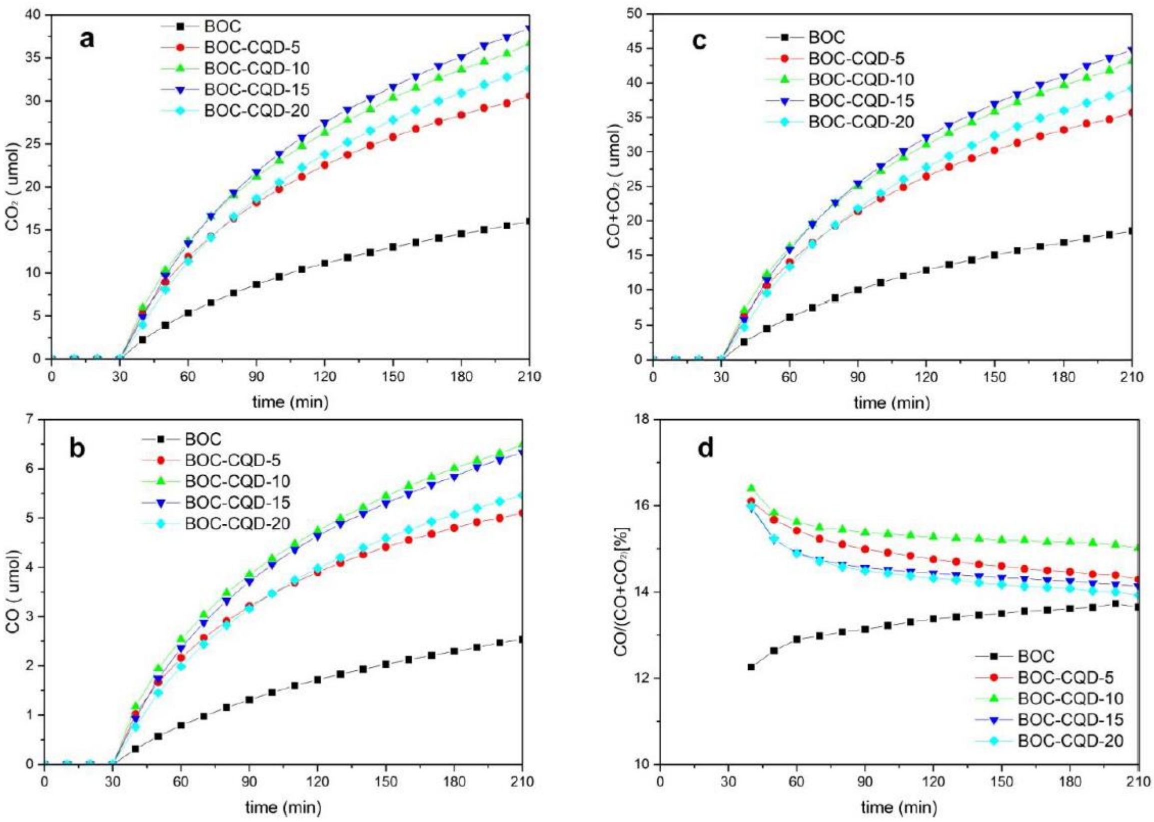

Figure S4. The CO_2 productivity **(a)**, the CO productivity **(b)**, the total productivity of CO_2 + CO **(c)**, the rate of CO_2/CO **(d)** of the as-prepared samples for toluene removal in air.

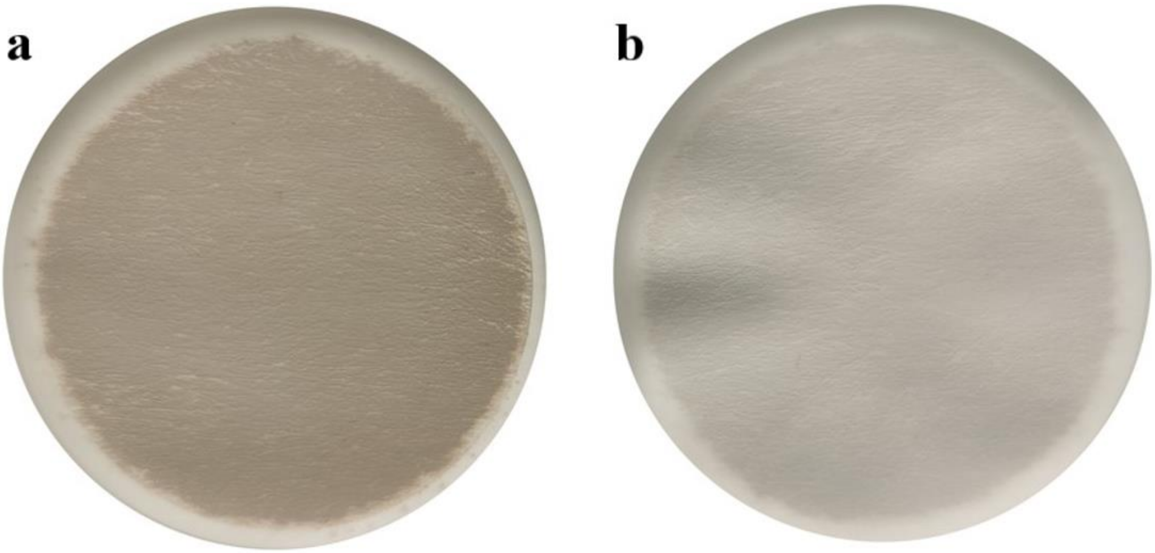

Figure S5. The photo images of BOC-CQD-15 under IR, Vis, UV, and full light irradiation during five cycles : before **(a)** and after **(b)**.
